# miRNA-142-3p functions as a potential tumor suppressor directly targeting FAM83D in the development of ovarian cancer

**DOI:** 10.18632/aging.203998

**Published:** 2022-04-22

**Authors:** Guangyu Gao, Xiaofei Guo, Wenyong Gu, Yufeng Lu, Zhigang Chen

**Affiliations:** 1 Department of Oncology, The Second Affiliated Hospital of Soochow University, Suzhou 215004, China; 2 Department of Ultrasound, The Second Affiliated Hospital of Soochow University, Suzhou 215004, China

**Keywords:** microRNA-142-3p, ovarian cancer, GEO, FAM83D, IHC

## Abstract

Background: FAM83D (family with sequence similarity 83, member D) is of particular interest in tumorigenesis and tumor progression. Ovarian cancer is the leading cause of cancer-related death in women all over the world. This study aims to research the association between FAM83D and ovarian cancer (OC).

Methods: The gene expression data of OC and normal samples (GSE81873 and GSE27651) was downloaded from Gene Expression Omnibus (GEO) dataset. The bioinformatics analysis was performed to distinguish two differentially expressed genes (DEGs), prognostic candidate genes and functional enrichment pathways. Immunohistochemistry (IHC), Quantitative Real-time PCR (qPCR), and luciferase reporter assays were utilized for further study.

Results: There were 56 DEMs and 63 DEGs in cancer tissues compared to normal tissues. According to the km-plot software, hsa-miR-142-3p and FAM83D were associated with the overall survival of patients with OC. Besides, Multivariate analysis included that hsa-miR-142-3p and FAM83D were independent risk factors for OC patients. Furthermore, qPCR demonstrated that miRNA-142-3p and FAM83D were differentially expressed in normal ovarian tissues (NOTs) and ovarian cancer tissues (OCTs). IHC results indicated that FAM83D was overexpressed in OCTs compared with NOTs. Last but not least, luciferase reporter assays verified that FAM83D was a direct target of hsa-miRNA-142-3p in OC cells.

Conclusions: The prognostic model based on the miRNA-mRNA network could provide predictive significance for the prognosis of OC patients, which would be worthy of clinical application. Our results concluded that miR-142-3p and its targets gene FAM83D may be potential diagnostic and prognostic biomarkers for patients with OC.

## INTRODUCTION

Ovarian cancer (OC) is one of the main causes of female death. 70% of patients are in the advanced stage (clinical stage III ~ IV), and peritoneal metastasis is the characteristic metastasis symptom of patients with advanced OC. Although the incidence rate is not high, the mortality rate is 2.9%, which is the top ten of female malignant tumor mortality. For OC, the current standard treatment is not optimistic [1]. One reason is that most patients are diagnosed only in the late stage of the disease. Standard treatment for OC usually includes surgery and platinum chemotherapy. Despite available treatments, the recurrence rate of OC is still high, and about 75% of advanced patients are incurable [2, 3]. Genomics research shows that there is no regulated gene mutation, and many clinical trials have failed to bring lasting clinical benefits [4]. Molecular typing of OC through molecular expression differences, exploring the microenvironment characteristics of different molecular subtypes of OC, and formulating accurate treatment may be a feasible strategy to improve the dilemma of OC treatment.

Since the discovery of lin-4 and let-7, the founding members of the microRNA (miRNA) family, hundreds of miRNAs have been found in viruses, plants, and animals by molecular cloning and bioinformatics methods [5]. There are more than 1000 known human miRNAs, which control more than 50% of mammalian protein-coding genes. MiRNAs can be overexpressed or inhibited in different diseases. It is a promising therapeutic research field to inhibit or replace microRNAs [6]. Although microRNA has only 20 nucleotides, it plays a key role in biological function by targeting plenty of mRNAs [7]. For example, one study found that miRNA-424-5p regulated ferroptosis by targeting Acyl-CoA Synthetase Long-Chain Family Member 4 in OC cells and indicated a potential therapeutic target for OC [8]. Wu et al. found that the downregulated microRNA-1301-3p inhabited lung carcinoma cell proliferation and migration and has a strong negative correlation with Polymerase I and transcript release factor [9]. Another study also indicated the expression level of miR-200-b control PD-L1 expression in lung carcinoma cells. Furthermore, it may function as a potential biomarker for PD-L1 expression in lung cancer patients [10]. These studies indicated that microRNAs may be related to the progression of cancer, and their mechanisms may take part in the pathogenesis of tumors via controlling cancer-associated genes.

In this article, DEGs and DEMs were selected by researching 1 OC mRNA microarray dataset and 1 miRNA dataset. We aimed to study the relationship between miR-142-3p, FAM83D (we identified), and the development of OC.

## RESULTS

### Identification of DEGs

**Figure 1:**
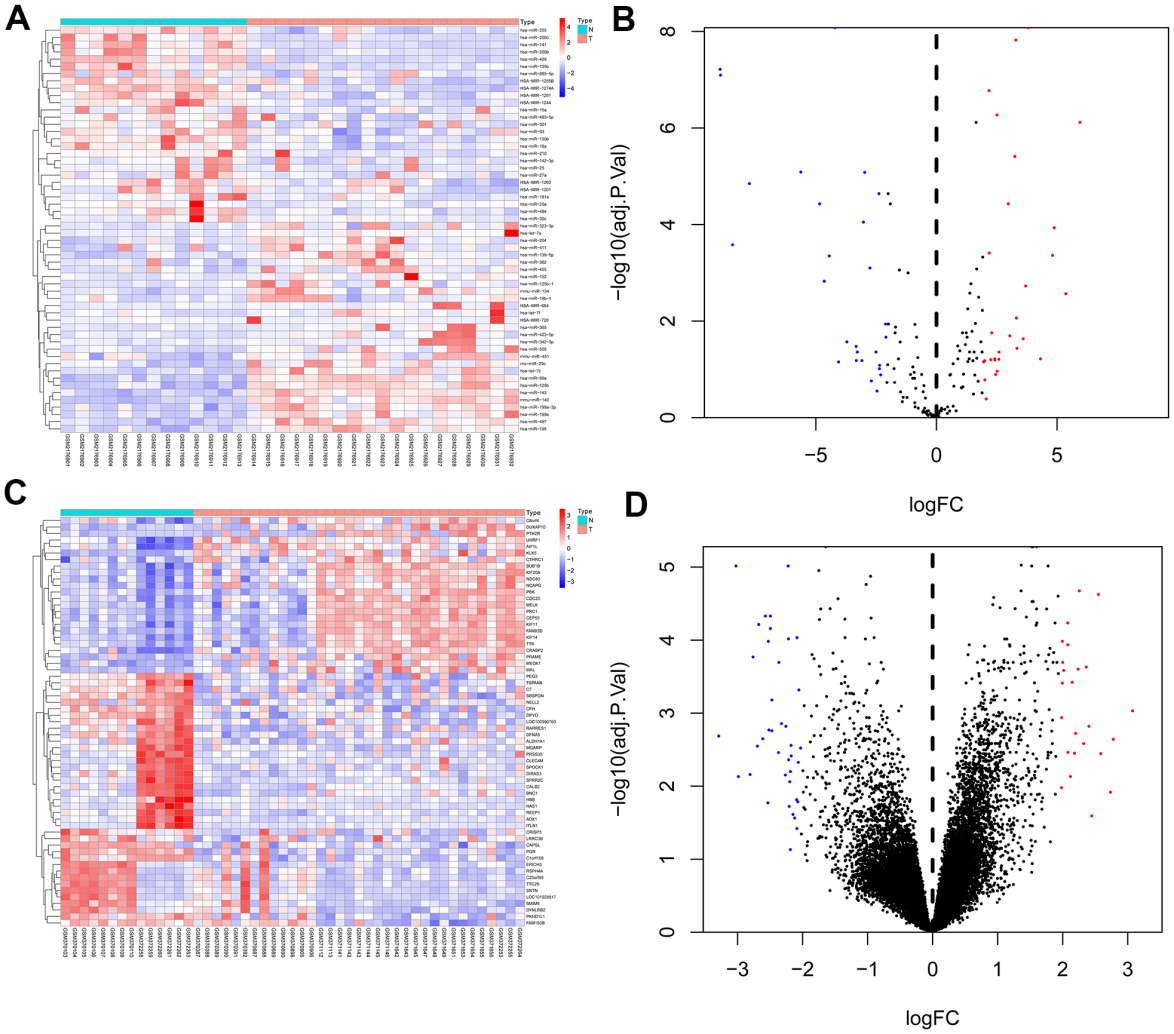
**Heat map and volcano map of differentially expressed genes of GSE81873 and GSE27651.** (**A**). Heat map of DEGs in GSE81873. (**B**). Volcano map of DEGs in GSE81873. (**C**). Heat map of DEGs in GSE27651. (**D**). Volcano map of DEGs in GSE27651. Red dots represent up-regulated genes and blue or green dots represent down-regulated genes.

We used GEO2R to analysis the DEMs and DEGs from the GSE81873 and GSE27651. Based on the cut-off criteria, 56 DEMs such as hsa-microRNA-142-3p, hsa-microRNA-429, hsa-microRNA-199a-3p, hsa-microRNA-484, hsa-microRNA-139-5p, hsa-microRNA-483-5p, hsa-microRNA-423-5p, hsa-microRNA-342-3p, hsa-microRNA-885-5p, and 63 DEGs were selected (Figure 1).

### Gene ontology enrichment analysis

**Figure 2:**
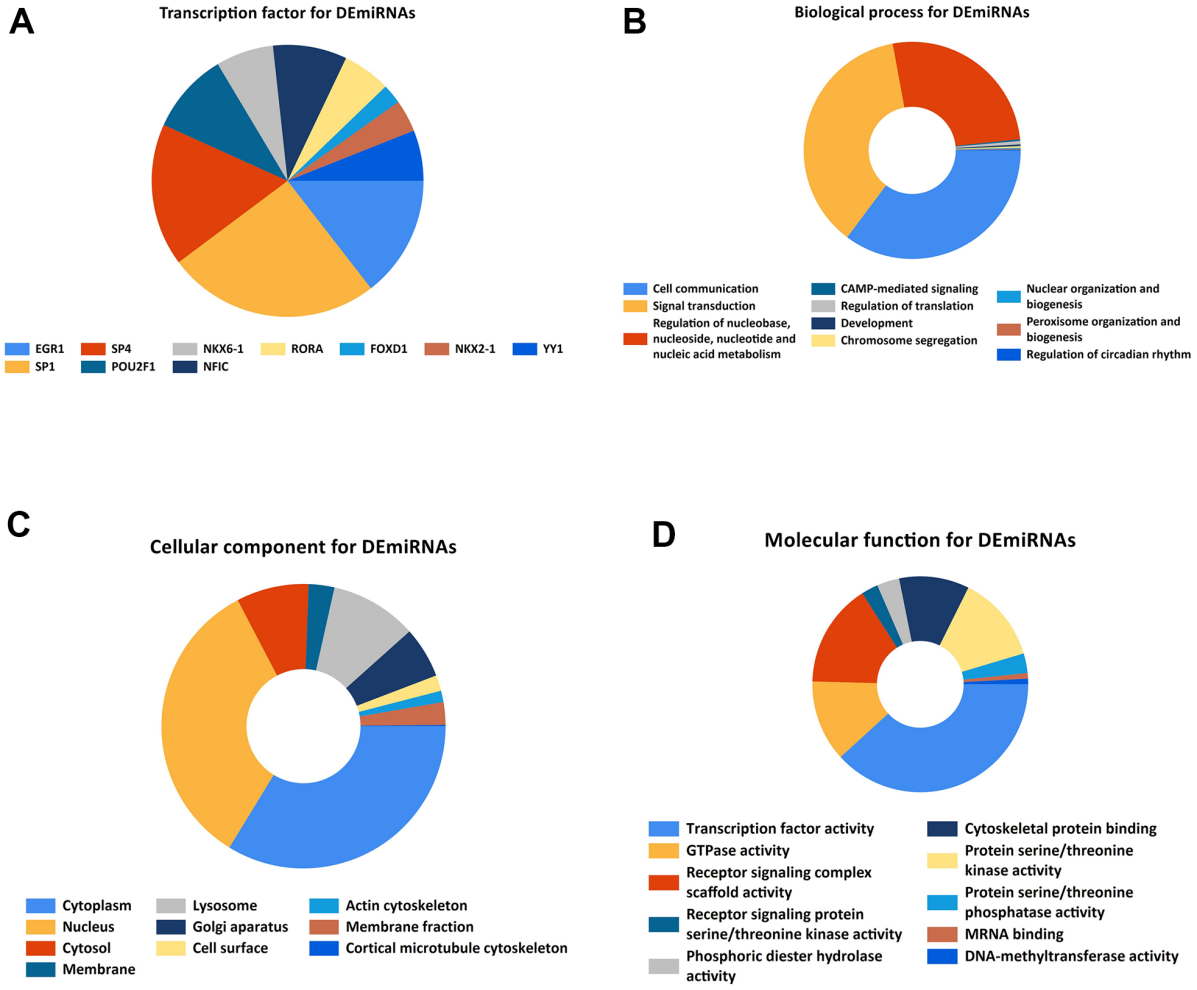
**Gene ontology enrichment.** (**A**). Identification of the potential transcription factors of DEMs by FunRich software. (**B**) biological process, (**C**) cellular component, and (**D**) molecular function enrichment analysis of the DEMs.

By utilizing FunRich and R software, we performed TF enrichment analysis (Figure 2A).

**Figure 3:**
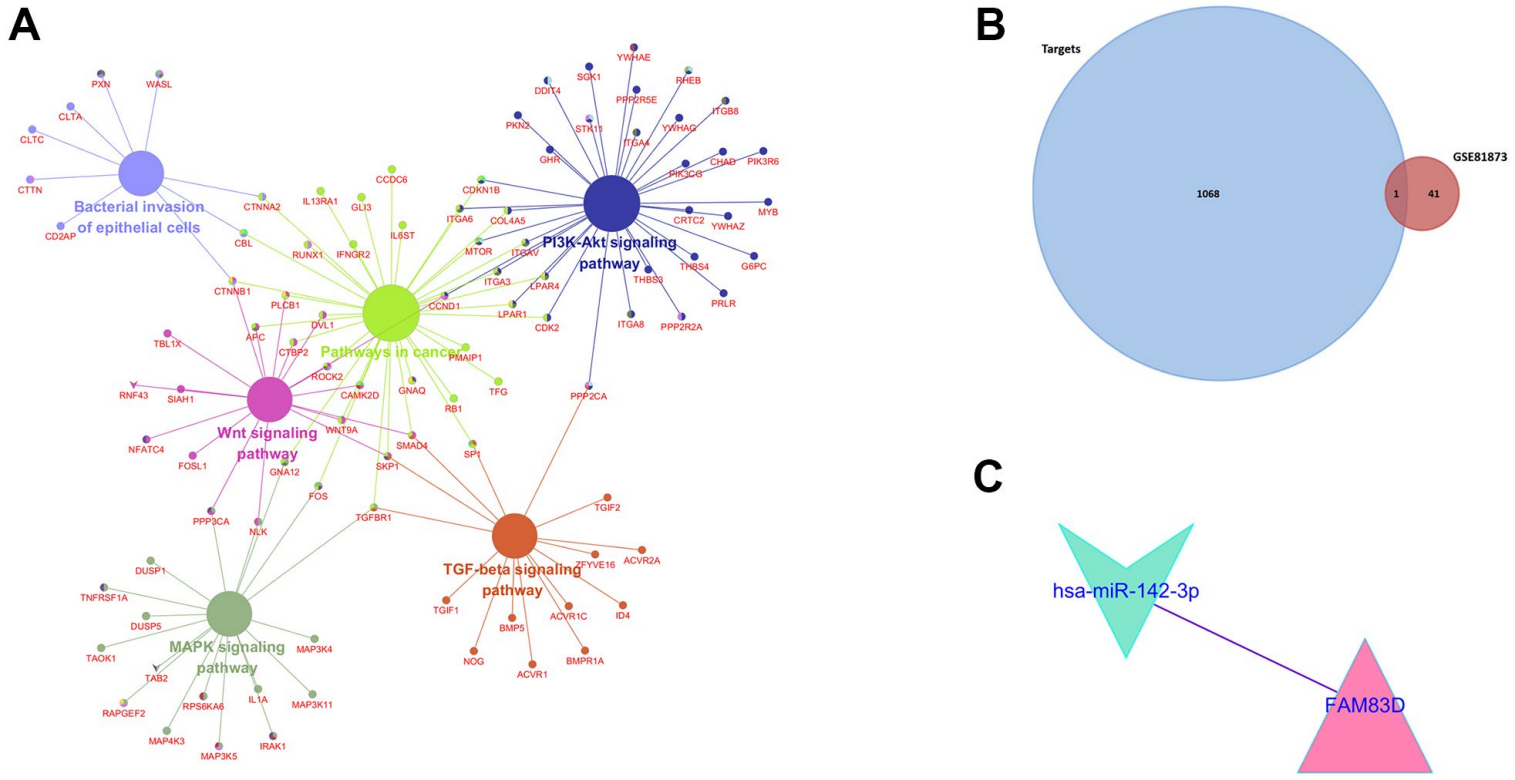
(**A**). KEGG pathway enrichment analysis of potential target mRNAs. (**B**). Venn Diagram of GSE81873 and GSE27651. (**C**). Identified target mRNAs and miRNA-mRNA regulatory network.

KEGG and GO function enrichment analyses were performed here. As for GO function analysis, three GO were selected: molecular function (MF), cellular component (CC), and biological process 156 (BP). Expression analysis showed that DEGs had the most uniquely enriched terms for Cell communication, Signal transduction, Regulation of nucleobase, nucleotide and nucleic acid metabolism, Cytoplasm, Nucleus, Cytosol, Membrane, Transcription factor activity, GTPase activity, Receptor signaling complex scaffold activity, Receptor signaling protein serine/threonine kinase activity, and CAMP-mediated signaling (Figure 2B–2D). Besides, DEMs were mainly enriched in 6 pathways: MAPK signaling pathway, PI3K-Akt signaling pathway, Wnt signaling pathway, TGF-beta signaling pathway, Pathways in cancer, and Bacterial invasion of epithelial cells (Figure 3A).

### miRNA-mRNA regulatory network

According to FunRich software, 1069 potential target mRNAs were obtained and only 1 of them showed different expression levels in GSE27651 (FAM83D). Based on the association between them, 1 essential microRNA-mRNA pair (microRNA-142-3p and FAM83D) was selected for the next research (Figure 3B, 3C).

### genes expression and their associations with OC overall survival

**Figure 4:**
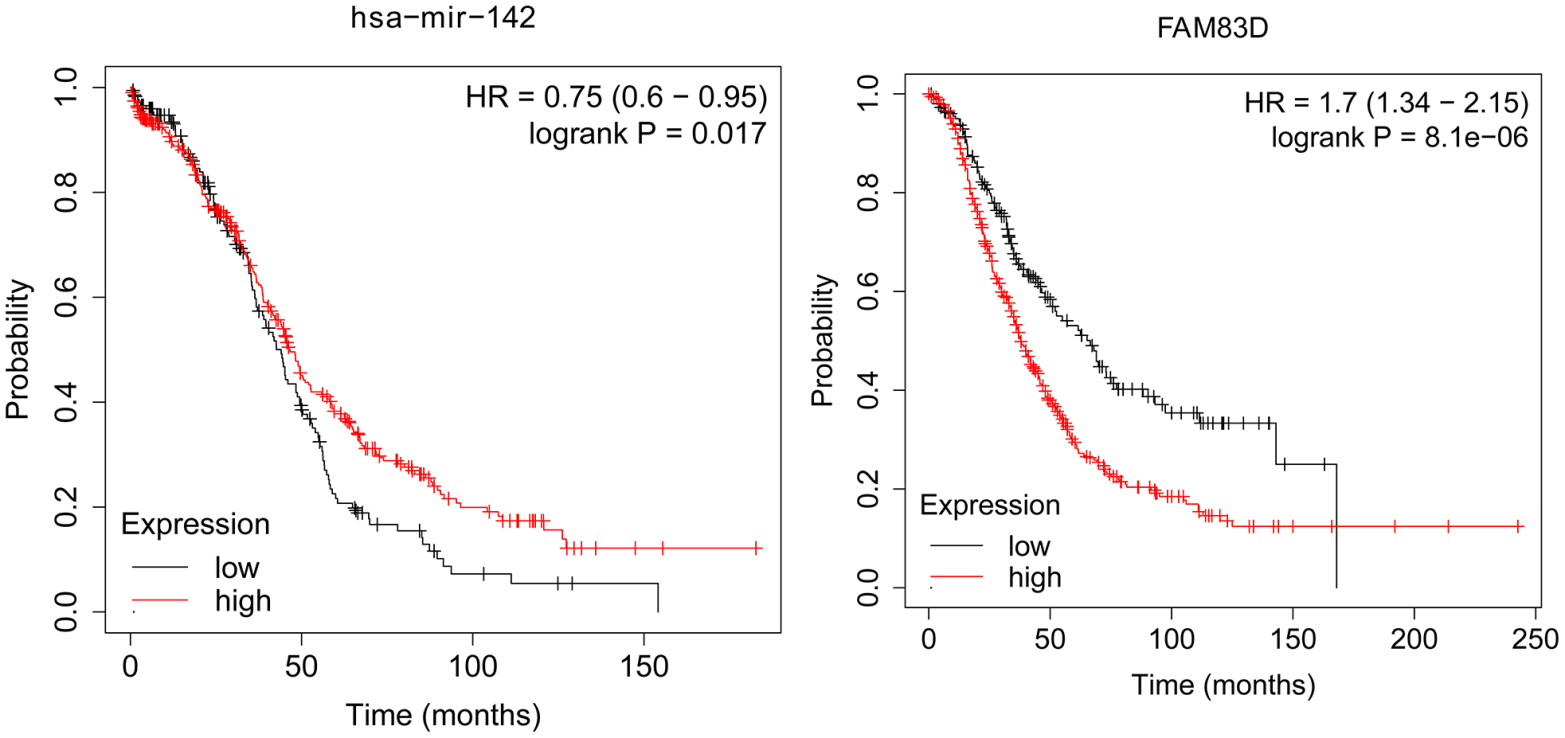
The association between the expression level of selected genes and overall survival of OC patients.

KM Plot was used to analyze the overall survival of patients with OC. According to uploading the miRNA and target gene we identified, we downloaded survival curves. The pictures showed that microRNA-124-3p and FAM83D were associated with the overall survival of patients with OC (Figure 4).

### Relationship between clinical characteristics and genes expression level of OC

Clinical and gene expression information of OC patients were downloaded in the TCGA database, such as patients’ age, race, FIGO stage, and primary therapy outcome (Tables 1, 2). FAM83D and miR-142-3p expression of patients with Stage III and Stage IV was higher than that of patients with Stage I and Stage II (P<0.05) according to the FIGO stage. Furthermore, associations between clinical characteristics and overall survival in OC were analyzed. According to medium FAM83D and miR-142-3p expression, patients were divided into two groups. Our results indicated that FAM83D mRNA and miR-142-3p expression level (P<0.05), FIGO stage (P<0.05), Primary therapy outcome (P<0.05), and tumor stage (P<0.05) was related to the OS of patients with OC (Tables 3, 4).

**Table 1:** Relationship between the expression level of FAM83D and clinical characteristics in OC.

**Characteristic**	**Low expression of FAM83D**	**High expression of FAM83D**	**p**
n	189	190	
FIGO stage, n (%)			**0.007**
Stage I	0 (0%)	1 (0.3%)	
Stage II	53 (13.9%)	10 (2.7%)	
Stage III	100 (26.3%)	155 (41.2%)	
Stage IV	35 (9.3%)	22 (5.9%)	
Primary therapy outcome, n (%)			0.484
PD	12 (3.9%)	15 (4.9%)	
SD	14 (4.5%)	8 (2.6%)	
PR	22 (7.1%)	21 (6.8%)	
CR	102 (33.1%)	114 (37%)	
Race, n (%)			0.840
Asian	5 (1.4%)	7 (1.9%)	
Black or African American	13 (3.6%)	12 (3.3%)	
White	161 (44.1%)	167 (45.8%)	
Age, n (%)			0.383
<=60	99 (26.1%)	109 (28.8%)	
>60	90 (23.7%)	81 (21.4%)	
Age, median (IQR)	60 (52, 68)	58 (50, 68)	0.390

**Table 2:** Relationship between the expression level of hsa-miR-142-3p and clinical characteristics in OC.

**Characteristic**	**Low expression of hsa-miR-142-3p**	**High expression of hsa-miR-142-3p**	**p**
n	248	248	
FIGO stage, n (%)			**0.009**
Stage I	0 (0%)	1 (0.2%)	
Stage II	13 (2.6%)	76 (15.3%)	
Stage III	199 (40.4%)	123 (24.8%)	
Stage IV	35 (7.1%)	45 (9.1%)	
Primary therapy outcome, n (%)			0.210
PD	21 (5.1%)	15 (3.7%)	
SD	16 (3.9%)	9 (2.2%)	
PR	24 (5.9%)	32 (7.8%)	
CR	139 (34.1%)	152 (37.3%)	
Race, n (%)			0.472
Asian	10 (2.1%)	6 (1.3%)	
Black or African American	14 (2.9%)	18 (3.8%)	
White	215 (44.9%)	216 (45.1%)	
Age, n (%)			0.786
<=60	139 (28%)	135 (27.2%)	
>60	109 (22%)	113 (22.8%)	
Age, median (IQR)	58.5 (51, 69)	59 (51.75, 68.25)	0.873

**Table 3:** Relationship between overall survival and the expression level of FAM83D researched by univariate and multivariate Cox regression.

**Characteristics**	**Total(N)**	**Univariate analysis**			**Multivariate analysis**	
		**Hazard ratio (95% CI)**	**P value**		**Hazard ratio (95% CI)**	**P value**
FIGO stage	374					
Stage I & Stage II	24	Reference				
Stage III	293	2.045 (0.905-4.621)	0.085		1.850 (0.670-5.108)	0.235
Stage IV	57	2.495 (1.057-5.889)	**0.037**		2.563 (1.541-4.517)	**0.041**
Primary therapy outcome	307					
PD	27	Reference				
SD	22	0.441 (0.217-0.895)	**0.023**		0.463 (0.222-0.967)	**0.040**
PR	42	0.652 (0.384-1.107)	0.113		0.637 (0.360-1.126)	0.121
CR	216	0.152 (0.093-0.247)	**<0.001**		0.203 (0.120-0.344)	**<0.001**
Race	364					
Asian & Black or African American	37	Reference				
White	327	0.637 (0.405-1.004)	0.052		0.738 (0.434-1.255)	0.262
FAM83D	377	1.645 (1.916-3.192)	**0.011**			
Tumor status	336					
Tumor free	72	Reference				
With tumor	264	9.576 (4.476-20.486)	**<0.001**		9.616 (3.875-23.866)	**<0.001**

**Table 4:** Relationship between overall survival and the expression level of hsa-miR-142-3p researched by univariate and multivariate Cox regression.

**Characteristics**	**Total(N)**	**Univariate analysis**			**Multivariate analysis**	
		**Hazard ratio (95% CI)**	**P value**		**Hazard ratio (95% CI)**	**P value**
FIGO stage	490					
Stage I & Stage II	30	Reference				
Stage III	380	2.179 (1.076-4.411)	**0.031**		1.513 (1.664-3.446)	**0.004**
Stage IV	80	2.785 (1.331-5.829)	**0.007**		1.333 (1.563-3.155)	**0.013**
Primary therapy outcome	407					
PD	36	Reference				
SD	25	0.453 (0.241-0.854)	**0.014**		0.452 (0.237-0.862)	**0.016**
PR	55	0.668 (0.426-1.047)	0.079		0.635 (0.400-1.010)	0.055
CR	291	0.168 (0.112-0.251)	**<0.001**		0.211 (0.139-0.319)	**<0.001**
Race	478					
Asian & Black or African American	48	Reference				
White	430	0.764 (0.513-1.138)	0.186			
hsa-miR-342-3p	494	0.835 (0.738-0.946)	**0.005**		1.910 (1.783-2.458)	**0.019**
Tumor status	442					
Tumor free	95	Reference				
With tumor	347	8.796 (4.784-16.170)	**<0.001**		8.362 (4.057-17.236)	**<0.001**

### Validation of the expression with qRT-PCR and IHC

**Figure 5:**
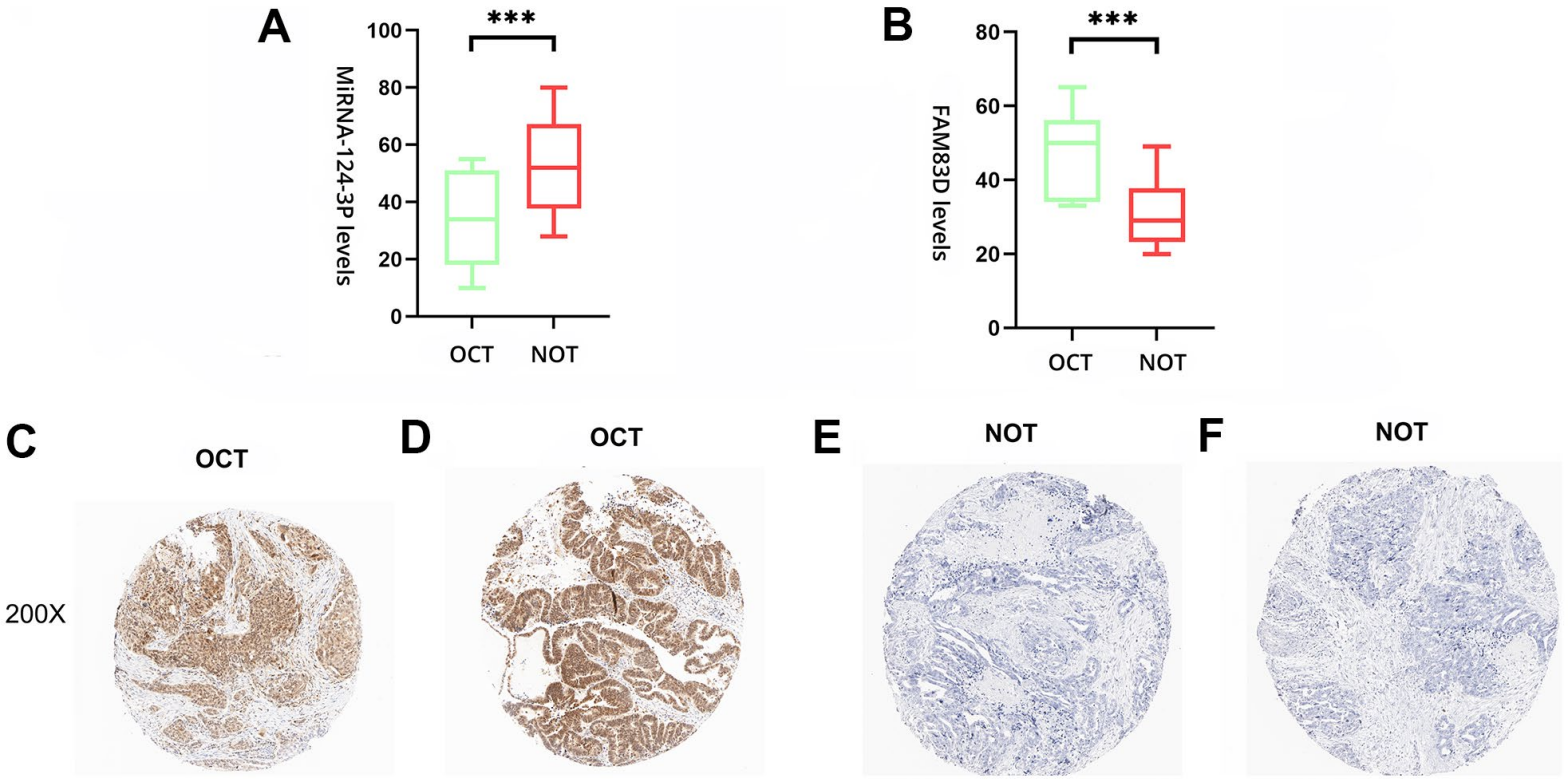
**The expression level of hsa-miR-142-3p and FAM83D in normal ovarian tissues (NOTs) and OC tissues (OCTs).** (**A**). Validation of hsa-miR-142-3p ((***, p < 0.001). (**B**). Validation of FAM83D (***, p < 0.001). (**C**, **D**). The expression level of FAM83D in human OCTs. (**E**, **F**). The expression level of FAM83D in human NOTs (scale bar: 200×).

To deeply evaluate the expression of miR-142-3p and FAM83D, 20 pairs of normal ovarian tissues (NOT) and OC tissues (OCT) were enrolled as a validation cohort. The qRT-PCR approach was utilized to verify the differential expression levels from the patient’s tissues. The same as the microarray data, hsa-miR-142-3p was significantly downregulated (Figure 5A) and FAM83D was overexpressed (Figure 5B) between 20 pairs of NOTs and OCTs which indicated that hsa-miR-142-3p and FAM83D could be the candidate biomarkers for OC. Besides, this expression level of genes was detected by IHC. The staining intensity of FAM83D was divided into 0, 1, 2, or 3, corresponding to colorless, light yellow, light brown, and squid ink. In addition, the percentage score is defined as: 0% to 5%, 0; 6% - 25%, 1 point; 26-50%, 2 points; 50-75%, 3 points; 76% to 100%, 4 points. The final histochemical score was calculated by multiplying the intensity score by the percentage score. The final staining scores were negative (0), low (6), and strong (≥ 6). Statistically, the overall expression of FAM83D was much higher in OC tissues than in the adjacent noncancerous tissues (P < 0.001) (Figure 5C–5F).

### Confirmation of the association between miR-142-3p and FAM83D

**Figure 6:**
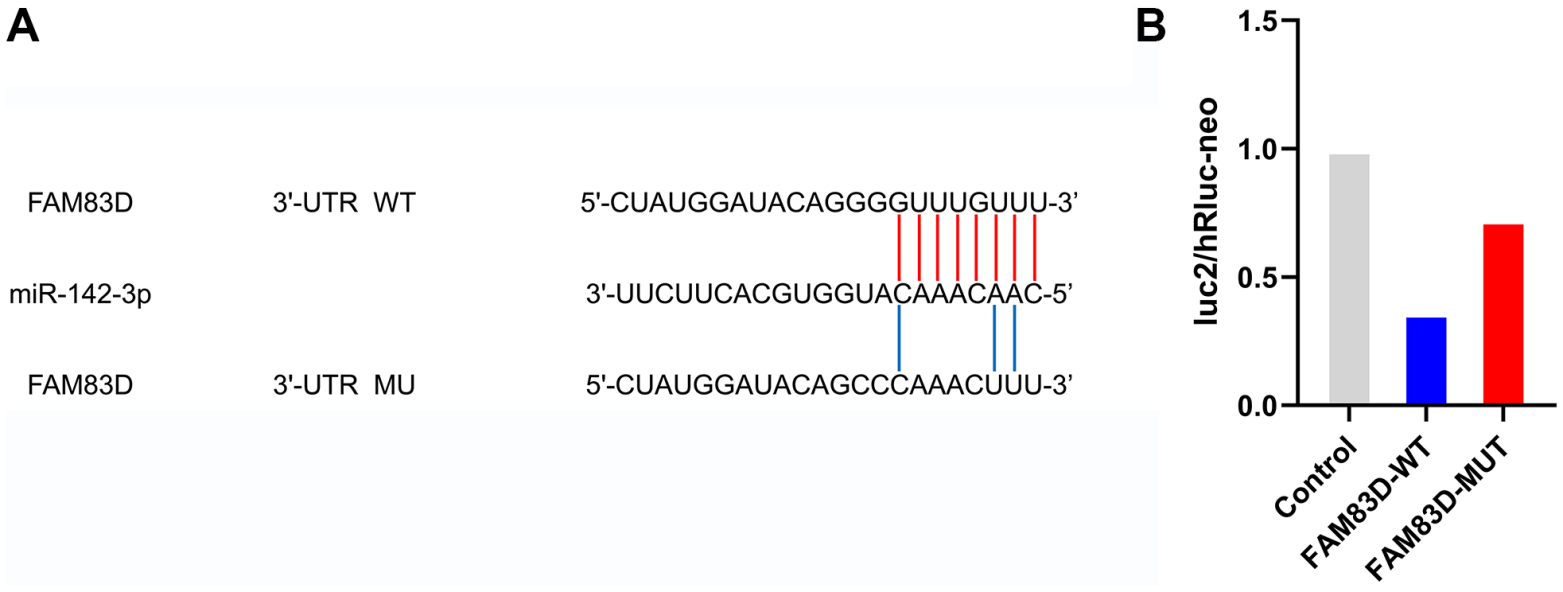
**FAM83D is a direct target of miR-142-3p in OC cells.** (**A**) Putative binding sites in FAM83D 3’UTR for miR-142-3p were predicted by bioinformatics analysis (microRNA.org). (**B**). Validation of luc2/hRluc-neo between miR-142-3p and FAM83D.

The Dual-Luciferase Reporter Assay System was utilized to evaluate the association between FAM83D and microRNA-142-3p. The FAM83D-WT activity of wild-type luciferase was 36.40%, and the FAM83D-MUT activity of mutant luciferase was 72.80%. These outcomes indicated that microRNA-142-3p reduced the luciferase activity of the wild-type FAM83D reporter gene (Figure 6).

## DISCUSSION

In 2020, there were 19.29 million new cancer cases worldwide, including 10.06 million males and 9.23 million females; In 2020, there were 9.96 million cancer deaths worldwide, including 5.53 million males and 4.43 million females [11, 12]. In this research, GSE81873 and GSE27651 were obtained in the GEO database. 56 DEMs and 63 DEGs were identified. For deeply understanding the process of the 56 DEMs in OC, FunRich and R software were utilized for further study. GO and KEGG analysis indicated that these DEMs were primarily associated with the Cytoplasm, Nucleus, and Transcription factor activity. Previous studies have shown that Lysosomes and Nucleus may function as a vital role in plenty of human diseases, such as tumors, obesity, and infection [13–15]. Furthermore, KEGG analysis showed that DEGs were mainly enriched in 6 pathways such as MAPK signaling pathway, PI3K-Akt signaling pathway, Wnt signaling pathway, TGF-beta signaling pathway, Pathways in cancer, and Bacterial invasion of epithelial cells, which were indicated to influence migration and proliferation [16]. The MAPK signaling pathway is a common signaling pathway closely associated with carcinoma, which will not be discussed here. Phosphatidylinositol-4,5-diphosphate-3-kinase (PI3K) is activated by many genes. In PI3K / Akt signal transduction pathway, phospholipid dependent kinase promotes the binding of protein kinase B (Akt) to the cell membrane. The phosphorylation of threonine and serine promotes the transfer of Akt from the cytoplasm to the nucleus and further mediates the biological effects of enzymes, including cell proliferation, inhibition of apoptosis, cell migration, vesicle transport, and cell carcinogenesis [17]. Besides, it can influence the epithelial-mesenchymal transition in plenty of methods to affect tumor aggressiveness [18]. As for OC, a previous study reported that it took an important part in OC tumorigenesis, proliferation and progression, and pre-clinical and clinical experience with several PI3K/AKT/mTOR pathway inhibitors [19]. Neurotrophins are produced by target tissues innervated by developing neurons, so these factors act on the end of axons and produce signals that must be transmitted back to the cell body [20]. It can activate the PI3K/Akt signal transduction pathway and other oncogenic signaling pathways [21]. For example, it has been confirmed that in the early stage of cancer, the increase of nerve density is parallel to the increase of neurotrophin level, but it remains to be clarified which cells in TME are the source of neurotrophin and the nature of the stimulants that initiate the production of neurotrophin [22]. Besides, a previous study reported that Neurotrophin Receptor TrkB (NTRK2) and Wnt β-Estradiol and MAPK signaling pathways are closely related to the worse prognosis of neuroblastoma [23]. Besides, the changes in neurotrophin signals are related to neurodegenerative diseases and mental diseases [24].

The regulated network was performed according to Cytoscape. 56 miRNAs (hsa-microRNA-142-3p, hsa-microRNA-429, hsa-microRNA-199a-3p, and hsa-microRNA-484) were selected for further research. After that, 1069 target genes were achieved and 1 of them showed a different expression level in GSE27651 (FAM83D). MicroRNA-142-3p encoding human chromosome 17q22 is a new tumor suppressor factor, which is observed in many tumors including breast carcinoma, hepatocellular carcinoma, bladder carcinoma, and ovarian carcinoma [25–30]. The downregulation of microRNA-142-3p is related to tumorigenesis via regulation, cell migration, cell apoptosis, and invasion through various signaling pathways. Besides, microRNA-142-3p has been found as a carcinogenic microRNA in human T-cell acute lymphoblastic leukemia by acting on glucocorticoid receptors and cyclic adenosine monophosphate/protein kinase A pathway [31]. Upregulated microRNA-142-3p also monitors the characteristics of breast carcinoma stem cells, at least in section by sensitizing the WNT signaling pathway and microRNA-150 expression [32]. A previous study reported that upregulated microRNA-142-3p inhibited colorectal cancer cell migration and invasion, indicating that microRNA-142-3p may act as an oncogene during colorectal cancer tumorigenesis [33]. As for OC, another study concluded that microRNA-142-3p suppressed the proliferation and chemoresistance of OC cells by targeting SIRT1. This indicates that microRNA-142-3p may be a therapeutic target for the cure of OC [27].

FAM83D (family with sequence similarity 83, member D) is a mitosis-related gene located on chromosome 20q11 [34]. Previous studies have indicated that FAM83D may be amplified and upregulated in a variety of tumors, including hepatocellular tumors [35], ovarian carcinoma [36], colorectal carcinoma [37], and lung adenocarcinoma [38]. In addition, researches have demonstrated that FAM83D may act as a carcinogenic role by suppressing the invasion and proliferation of hepatocellular carcinoma and inhibiting the cell cycle of lung adenocarcinoma by suppressing FBXW7 in breast carcinoma [39–41]. These outcomes indicated that FAM83D may be widely taken part in process of tumors. Besides, FAM83D can advance epithelial-mesenchymal transition and metastasis of non-small cell lung carcinoma cells through the AKT/mTOR signal pathway, also improve the sensitivity of NSCLC cells to cisplatin [42]. Furthermore, FAM83D is highly expressed in invasive epithelial ovarian cancer and is related to tumor stage and grade [43]. Last but not least, FAM83D can also promote ovarian carcinoma cell invasion and proliferation, while suppressing autophagy through the PI3K/AKT/mTOR signaling pathway [44]. Therefore, we selected and tested whether microRNA-142-3p and FAM83D were differently expressed between NOTs and OCT.s qPCR results indicated that microRNA-142-3p and FAM83D were differentially expressed in NOTs and OCTs. Besides, IHC results indicated that FAM83D had a significant difference in OCTs compared with NOTs.

Many studies have shown that the abnormal expression of miRNAs is caused by gene aberrations (including genetic and epigenetic changes) of many cancer types, and plays a role in the occurrence and development of cancer through the imbalance of target gene expression. Therefore, many miRNAs and their target genes are closely related to the pathogenesis of tumors, including cell proliferation, cell survival, and cell invasion. Our study indicated that plenty of DEGs and DEMs were taken part in the process of OC by some pathways and had prognostic value. Therefore, suppression of FAM83D and upregulated miR-142-3p may have latent remedy worth in OC patients.

## CONCLUSIONS

Our study indicated some reasons for the procession of OC. Plenty of DEMs and DEGs were selected between OC tissues and normal ovarian tissues. Besides, miR-142-3p and FAM83D were selected as latent biomarkers of OC. qPCR and IHC results indicated that microRNA-142-3p and FAM83D were differentially expressed in OC tissues. Besides, luciferase reporter assays verified that FAM83D was a direct target of miR-142-3p in OC cells. However, we need more cell experiments to prove it.

## MATERIALS AND METHODS

### Microarray data

The RNA-seq data of OC samples and corresponding normal ovarian tissues were retrieved from the GEO dataset (https://www.ncbi.nlm.nih.gov/geo/). The datasets of GSE81873 and GSE27651 were downloaded and divided into two groups.

### Differently expressed miRNAs research

GEO2R is software for differential analysis of expression microarray based on the GEO database.

Limma R package was used to identify DEGs in the construction cohort. The screening standards of DEGs for functional enrichment analysis were |log2FC|> 1 and FDR<0.05.

### Gene ontology and pathway enrichment analysis

Transcription factors (TF), Kyoto Encyclopedia of Genes and Genomes (KEGG), and Gene Ontology (GO) enrichment analyses of the DEGs were performed by using R clusterProfiler package, including the package of "GOplot”, “ggplot2”, “stringi”, “colorspace” and “digest”. Then, the pathway and process enrichment analyses were carried out by using Cytoscape.

### MicroRNA-mRNA regulatory network

At present, there are two generally recognized miRNA mechanisms: miRNA-mediated mRNA translation inhibition and miRNA-mediated mRNA-specific cleavage. In addition, researchers also found that miRNA may have other regulatory mechanisms, such as regulating the localization or stability of target mRNA, or acting on target molecules other than mRNAs, such as complementary binding with regulatory non-coding RNA or even miRNA, or competing with other RNAs to bind proteins to achieve its regulatory function. DEMs were uploaded to the FunRich software to achieve target mRNAs. Furthermore, GSE27651 was researched by utilizing R software. Based on the prediction conclusions of target mRNAs in FunRich software and the differentially expressed mRNAs of GSE27651, the microRNA-mRNA network was constructed.

### The association between the expression level of identified genes and overall survival of patients with OC

Kaplan Meier plotter can assess the impact of 54K (mRNA, miRNA, protein) on the survival rate of 21 types of cancer (including breast cancer (n = 6234), ovarian cancer (n = 2190), lung cancer (n = 3452) and gastric cancer (n = 1440). The sources of the Kaplan Meier plotter database include GEO, EGA, and TCGA. The main purpose of the tool is the discovery and validation of survival biomarkers based on a meta-analysis. In this study, patients with OC were divided into two groups. By uploading the DEGs we identified, corresponding survival curves were obtained.

### DEGs expression and clinical characteristics in the cancer genome atlas

The associated statistics offered by The Cancer Genome Atlas. The data of 1037 patients with ovarian cancer were downloaded in the TCGA database. The expression level of mRNAs, clinicopathological information, and general information of patients with OC were achieved.

### Immunohistochemical staining

20 pairs of OC tissues were prepared by buffering with 10% formalin for 24 hours. The study was approved by the Ethics Committee of the Second Affiliated Hospital of Soochow University and informed consent was obtained from all patients. 2 consecutive 5-m sections were taken from each formalin-fixed paraffin-embedded block and mounted on the glass slide treated with aminoalkyl silane, dewaxing with xylene, passing through graded alcohol, and then continue rinsing in deionized water and phosphate-buffered saline. It was blocked by 3% non-immune horse serum. The sections were incubated with broad-spectrum anti-cytokeratin AE1 / 3 (Dako, Santa Barbara, CA, USA) in 1:50 dilution overnight at room temperature. After washing twice in buffer, an appropriate biotinylated secondary antibody was applied for 30 minutes. After two more washes in the buffer, appropriate biotinylated secondary antibodies were applied for 30 minutes. The sections were developed under the microscope in Tris HCl buffer (pH 7.4) and 0.03% hydrogen peroxide for 20 minutes. Application of light Mayer hematoxylin. The expressions of FAM83D and β - Catenin were detected by the Chi-square test or Fisher exact test. GraphPad Prism 9 software was utilized for statistical analysis. P-value < 0.05 was considered statistically significant.

### Real-time quantitative polymerase chain reaction

Total RNA was extracted from OC tissues and normal tissues by utilizing TRIzol reagents (Invitrogen, Carlsbad, CA, USA) as per the manufacturer’s instructions. The total RNA was reverse-transcribed into cDNA by utilizing a PrimeScript™RT kit with gDNA Eraser (TaKaRa, China). We also constructed Quantitative Real-time PCR (qRT-PCR) by using SYBR Select Master Mix for CFX (Invitrogen) and the CFX Connect Real-Time PCR System (BioRad). The amplification conditions are 95° C for 15s, then 40 cycles, 95° C for 5S, 60° C for the 30s. Primer sequences of FAM83D were as follows: primer F, 5’- GCACTTCCCTTTGTTGTAGTC −3’, primer R, 5’-AGCACTTCCCTTAGGTTACTC −3’. Using glyceraldehyde-3-phosphate dehydrogenase (GAPDH) as endogenous control, the recorded data were analyzed and processed by 2−ΔΔCt.

### Luciferase reporter assay

The relationship between miR-142-3p and FAM83D was verified by performing a luciferase reporter assay. To construct a luciferase ratio vector, we amplified the wild or mutant fragment FAM83D 3 ‘- UTR containing the hypothetical binding site miR-142-3p and subcloned it into luciferase pLUC vector (Wuhan, Ruibo, China). OC cells were co-transfected with pLUC-FAM83D 3 ‘- UTR wild (WT) or pLUC-FAM83D 3’ - UTR (MU) mutants and miR-142-3p or NC analogues using Lipofectamine 2000 according to the manufacturer’s instructions. After 48 hours of transfection, the relative activities of luciferase and double luciferase were detected by the analytical system (Promega, Madison, WI, USA). The luciferase activity of fireflies was normalized to *Renilla* luciferase activity.

### Statistical methods

The data were expressed as mean ± standard error. All data analyses were performed using R software (version 3.6.6) and GraphPad Prism 9 software package (GraphPad Software, Inc., La Jolla, California, USA). The differences between the two addiction groups were compared by student’s t-test. All the experiments were made in triplicate.

### Availability of data and materials

The datasets used and/or analyzed during the current study are available from the corresponding author on reasonable request.

### Ethics approval and consent to participate

The study was conducted according to the guidelines of the Declaration of Helsinki, and approved by the Ethics Committee of the Soochow University.
